# Classification system of the tibiofibular syndesmosis blood supply and its clinical relevance

**DOI:** 10.1038/s41598-018-28902-z

**Published:** 2018-07-12

**Authors:** Izabela Mróz, Piotr J. Bachul, Krzysztof A. Tomaszewski, Tomasz Bereza, Krzysztof Gil, Jerzy A. Walocha, Artur Pasternak

**Affiliations:** 1 Department of Anatomy, Jagiellonian University Medical College, Krakow, Poland; 2 Department of Surgery, University of Chicago, Chicago, IL United States; 3 Department of Pathophysiology, Jagiellonian University Medical College, Krakow, Poland

## Abstract

Due to the lack of anatomical studies concerning complexity of the tibiofibular syndesmosis blood supply, density of blood vessels with further organization of syndesmotic vascular variations is presented in clinically relevant classification system. The material for the study was obtained from cadaveric dissections. We dissected 50 human ankles observing different types of arterial blood supply. Our classification system is based on the vascular variations of the anterior aspect of tibiofibular syndesmosis and corresponds with vascular density. According to our study the mean vascular density of tibiofibular syndesmosis is relatively low (4.4%) and depends on the type of blood supply. The highest density was observed among ankles with complete vasculature and the lowest when lateral anterior malleolar artery was absent (5.8% vs. 3.5%, respectively). Awareness of various types of tibiofibular syndesmosis arterial blood supply is essential for orthopedic surgeons who operate in the ankle region and radiologists for the anatomic evaluation of this area. Knowledge about possible variations along with relatively low density of vessels may contribute to modification of treatment approach by the increase of the recommended time of syndesmotic screw stabilization in order to prevent healing complications.

## Introduction

Tibiofibular syndesmosis is a fibrous connection localized between the fibular notch of the tibia and medial surface of the lateral ankle. It is stabilized by two ligaments: anterior and posterior tibiofibular ligament. Some authors distinguish three ligaments stabilizing this joint: anterior, posterior and interosseous tibiofibular ligament^1–4^. There is a lack of studies presenting the complexity of its arterial blood supply. Only McKeon *et al*. provided detailed anatomical description of the vessels supplying syndesmotic area^5^. However, this study is focused mainly on the supply of the anterior aspect of the tibiofibular syndesmosis with the consideration of the caliber of the vessels. Huber (1941) in his study mentioned a perforating (anterior) branch of the fibular artery (known in the anatomical nomenclature as peroneal artery), which pierces the interosseous membrane and runs across the anteroinferior region of the tibiofibular syndesmosis^6^. Bartonicek also reported a perforating branch of the peroneal artery that penetrates the interosseous membrane^1^. According to most studies the popliteal artery gives rise to anterior tibial artery and common tibiofibular trunk, which divides into posterior tibial artery and fibular artery. In most cases origin of the fibular artery is described as just below the level of the neck of fibula, about 5 cm below the bifurcation of the popliteal artery. Fibular artery in its distal part gives rise to anterior branch, posterior branch and communicating branch joining posterior tibial artery, and then continues its passage, finally spreading into the calcaneal branches. In general, three main arteries of the leg may have contribution in tibiofibular syndesmosis blood supply: posterior tibial, anterior tibial and fibular artery^7–11^. Understanding the anatomical variations in blood supply of tibiofibular syndesmosis is crucial for orthopedic surgeons operating in the ankle region and may contribute to the modification of the surgical technique to avoid complications as a direct consequence of accidental vessel injury. Among the most common complications is the malreduction resulting with decreased joint motion and chronic pain, both can lead to long-term disability. Another less prominent complication with yet poorly understood pathophysiology is heterotopic ossification. It can lead to ankle synostosis, resulting in pain and abnormal ankle kinematics^12^. Injury of the tibiofibular syndesmosis is associated with slower healing time in comparison with injuries of other ankle ligaments. Typically it results in significantly longer time away from ambulation and sport activities. The limited blood supply is the commonly recognized factor which may have a negative impact on healing of number of anatomical structures. Knowledge about this limitation is essential at the time of preoperative planning and selection of the potentially most successful treatment^5,13–17^. Insufficient vascular supply to an area of injury usually leads to delay in the healing process and increased overall morbidity^5^. The management of injury to the distal tibiofibular syndesmosis remains controversial in the treatment of ankle fractures. Operative fixation usually involves the insertion of a metallic diastasis screw. There are a variety of options for the position and characterization of the screw, the type of cortical fixation, and whether the screw should be removed prior to weight-bearing^18^. This paper reviews the relevant anatomy, the clinical and radiological diagnosis and the mechanism of trauma and alternative methods of treatment for injuries to the syndesmosis. The complex nature of vascular anatomy of the distal tibiofibular syndesmosis results with variation and disagreement in treatment strategies. The aim of this study is to present the complexity of the tibiofibular syndesmosis blood supply, further organization of its vascular variations into classification system and examination of vascular density of this region with clinical correlation.

## Results

**Figure 1:**
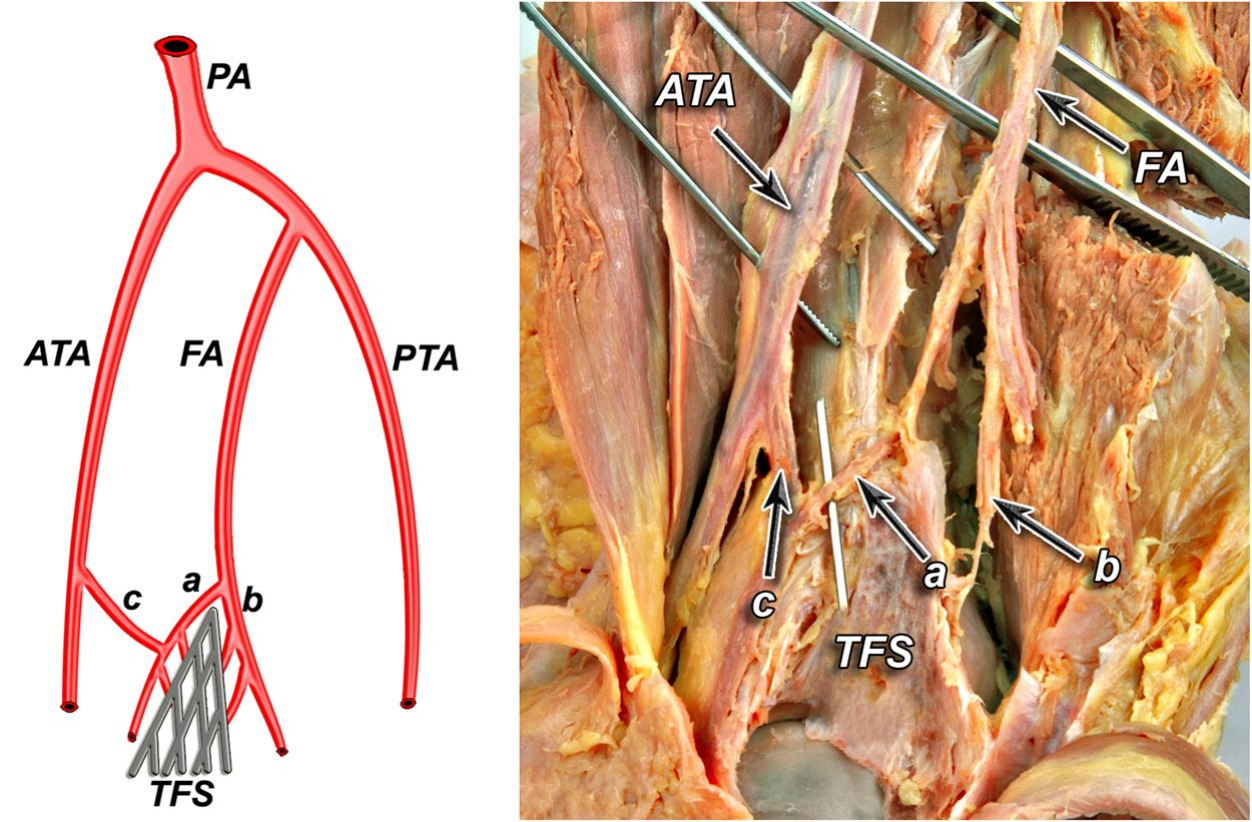
Anterior aspect of syndesmosis supplied by lateral anterior malleolar artery from anterior tibial artery and by anterior branch of fibular artery. Type IA – lateral anterior malleolar artery present and supplies anterior aspect of syndesmosis indirectly connecting to the anterior branch of fibular artery (52%).

**Figure 2:**
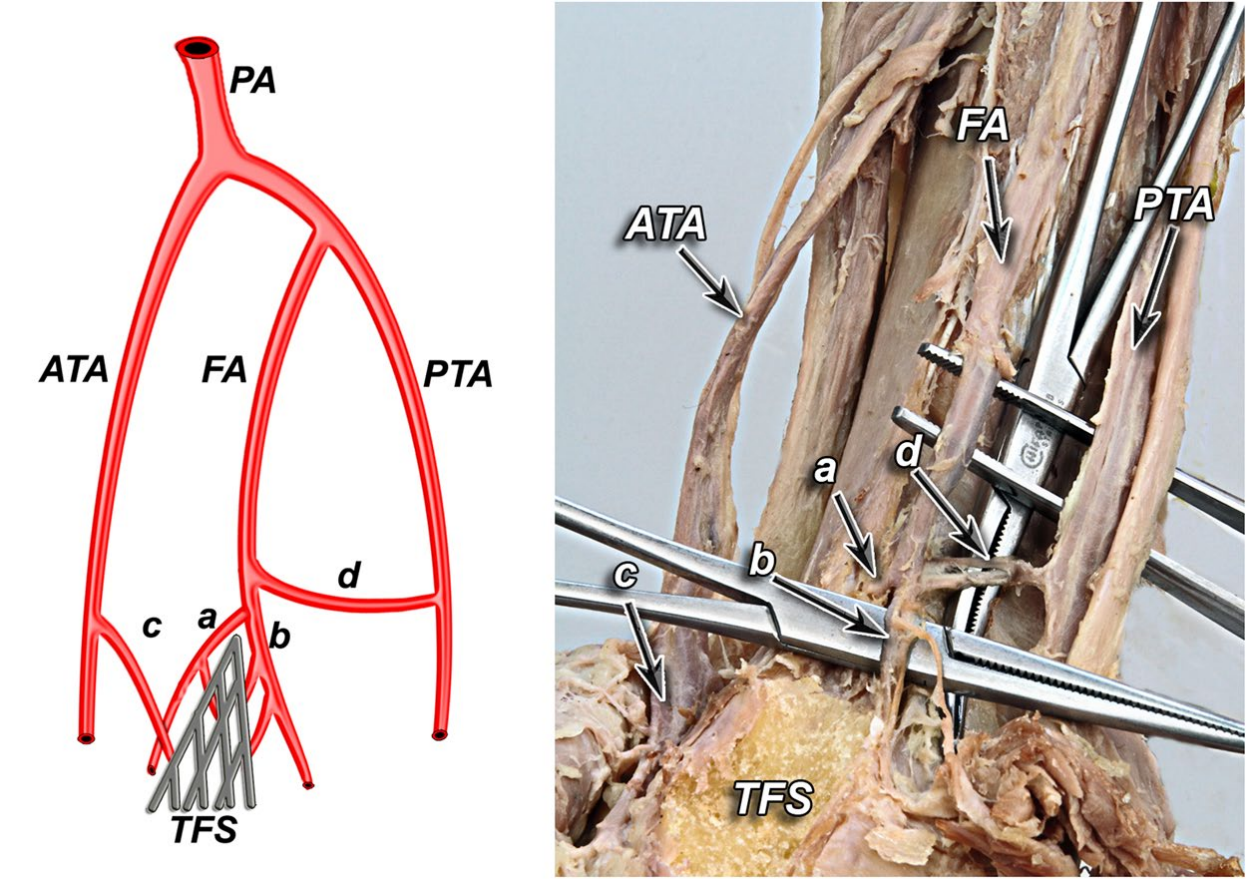
Type IB – lateral anterior malleolar artery present and supplies anterior aspect of syndesmosis directly (16%).

**Figure 3:**
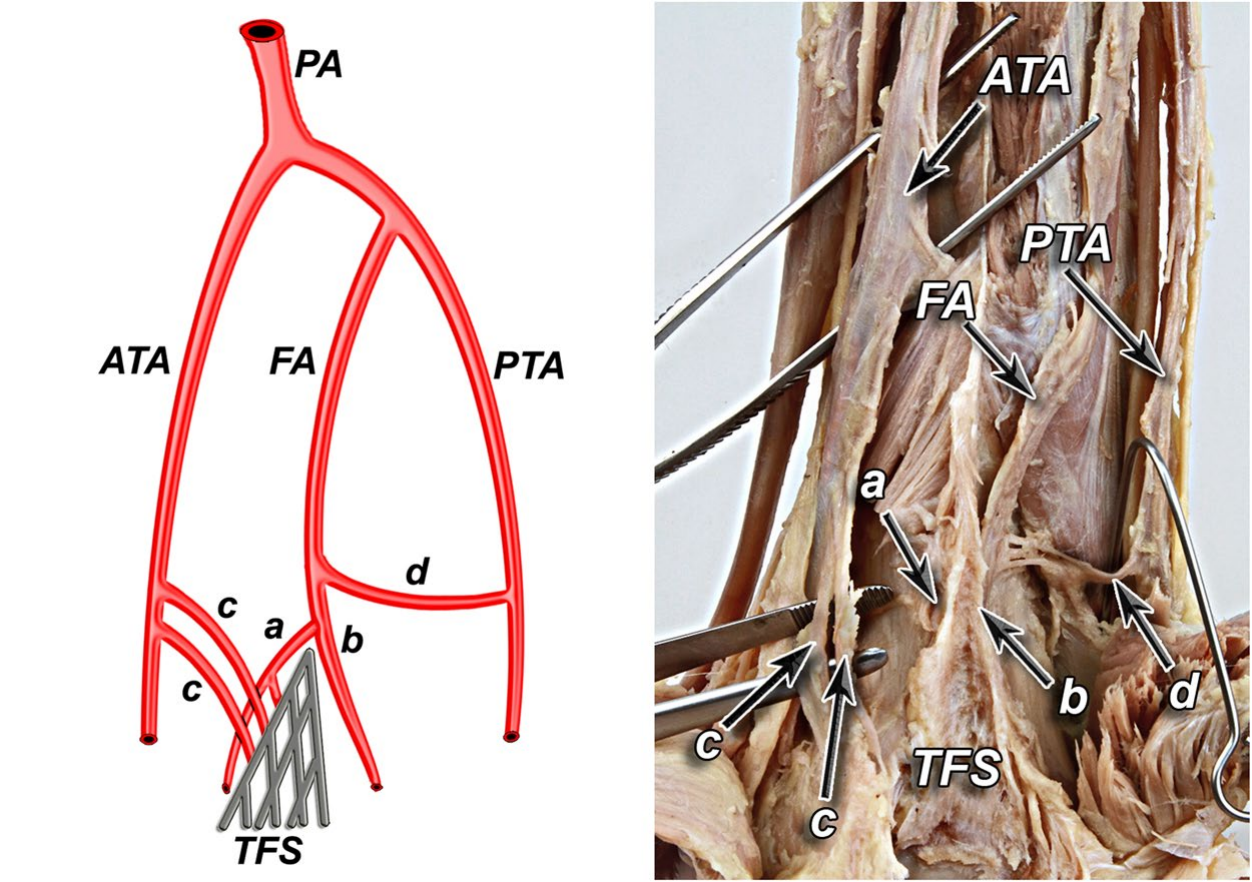
Type IB – lateral anterior malleolar artery present and supplies anterior aspect of syndesmosis directly (16%).

**Figure 4:**
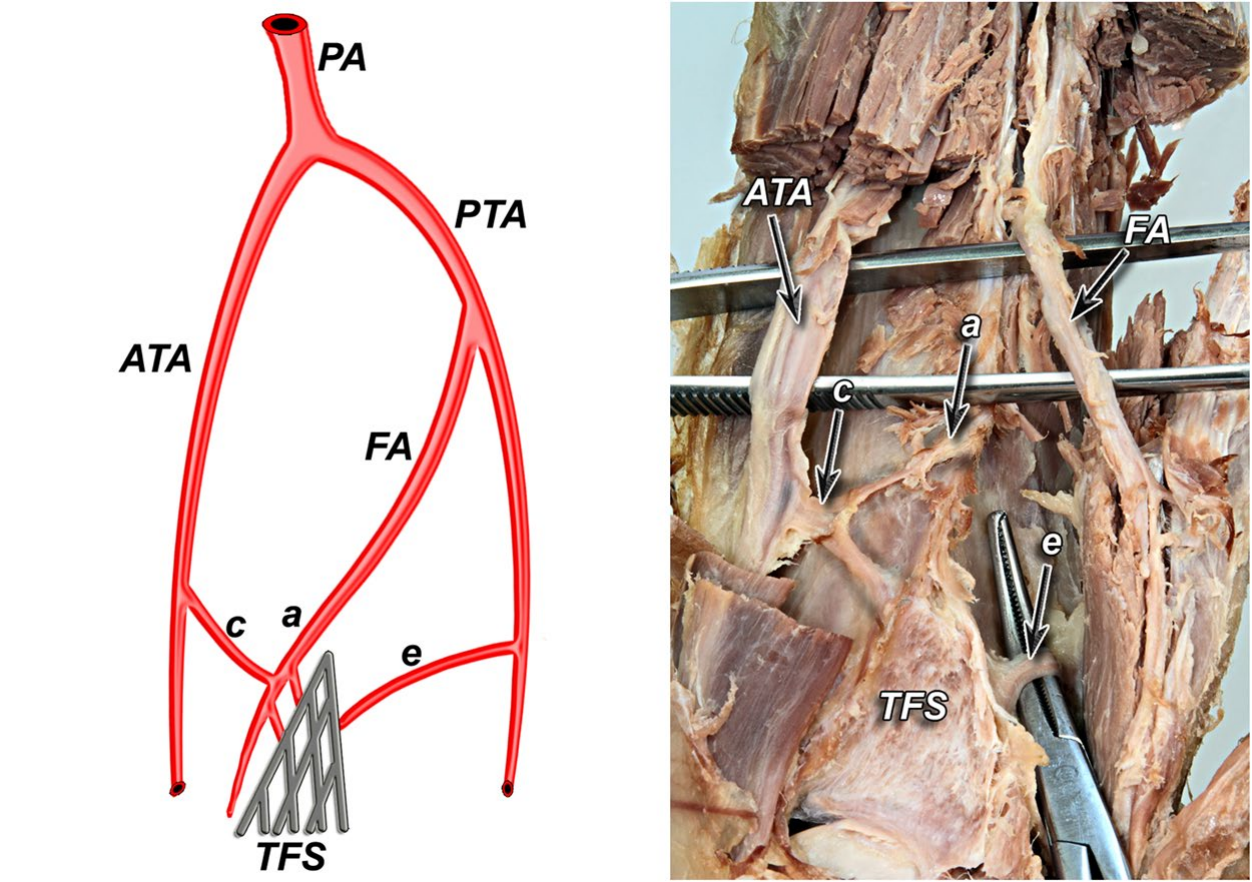
Type IC – Type IA or IB with coexisting anatomical variation of blood supply to posterior aspect of tibiofibular syndesmosis (4%).

**Figure 5:**
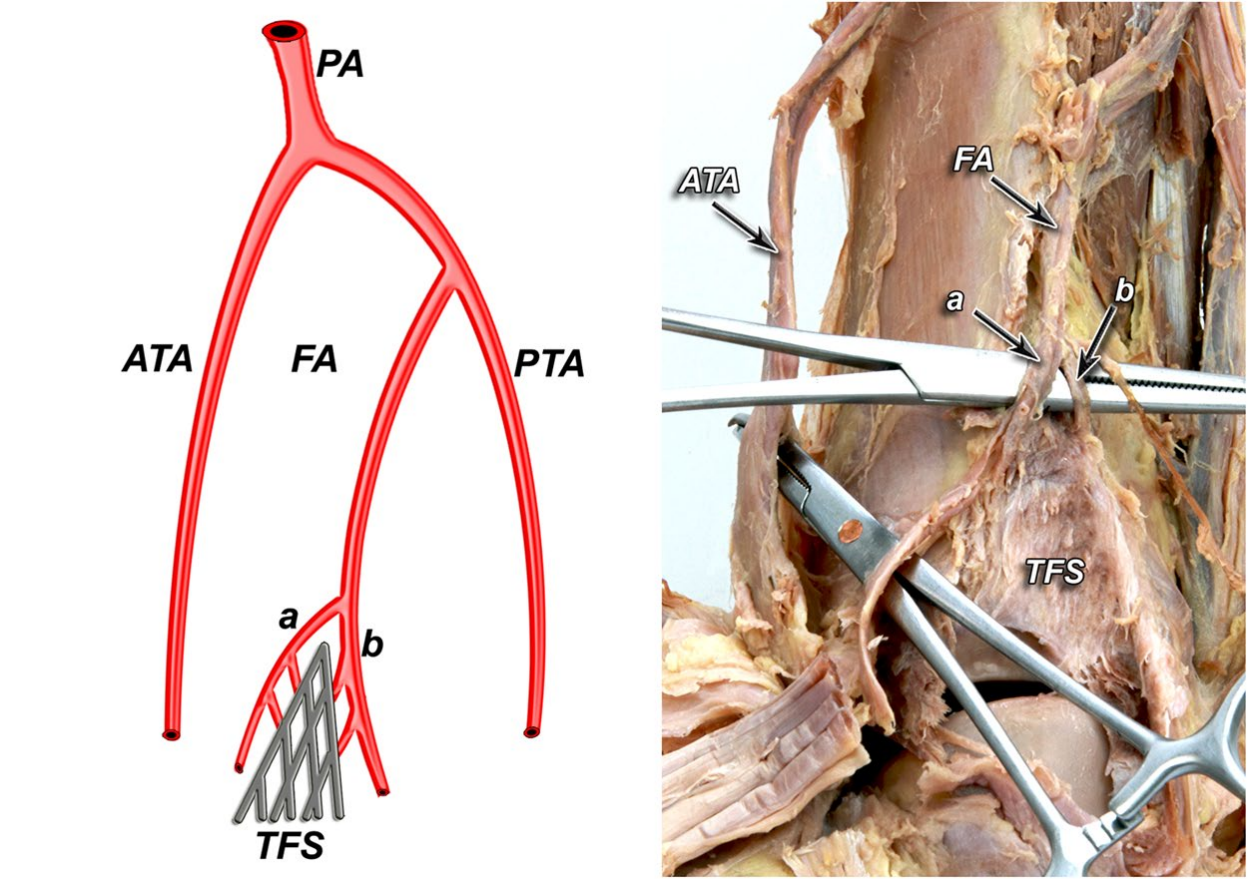
Type IIA – lack of lateral anterior malleolar artery but presence of anterior tibial artery (24%).

**Figure 6:**
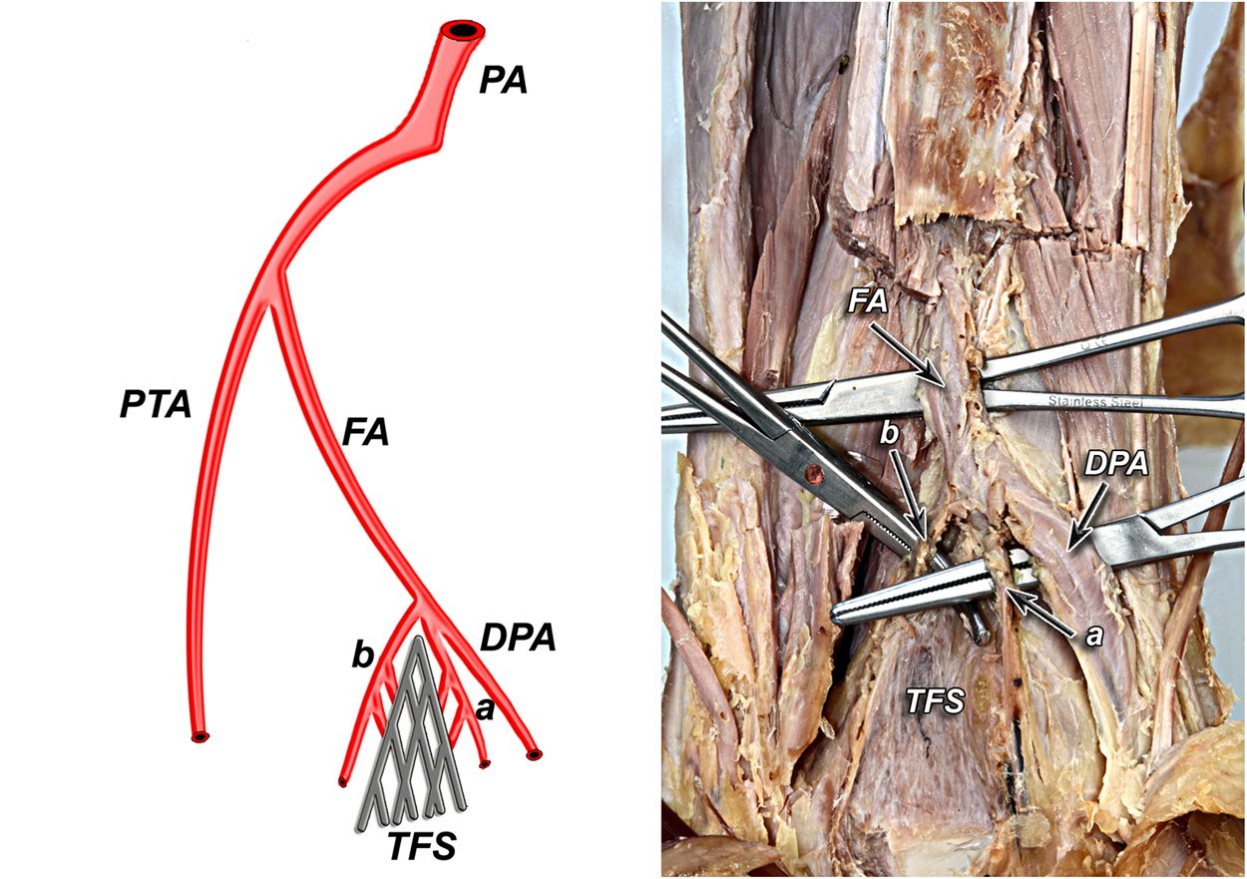
Type IIB – lack of anterior tibial and lateral malleolar artery (4%).

According to our observation all of the anatomical variants of tibiofibular syndesmosis blood supply can be divided into two types based on the presence of the lateral anterior malleolar artery, which is the branch of anterior tibial artery. It is in the opposite to the blood supply of the posterior aspect of the syndesmosis, which was rather constant in our study. Based on the literature^5^ and our findings, variants of tibiofibular syndesmosis blood supply were organized into unified and clinically useful classification system (Table 1, Figs 1–6). Our classification is based on the vascular variations of the anterior aspect of the syndesmosis and correlates with the density of syndesmotic vessels.

**Table 1:** The classification system of tibiofibular syndesmosis blood supply based on the presence of the lateral anterior malleolar artery.

**Type I** (**72%**) Anterior aspect of syndesmosis supplied by lateral anterior malleolar artery from anterior tibial artery and by anterior branch of fibular artery	**Type IA** – lateral anterior malleolar artery present and supplies anterior aspect of syndesmosis **indirectly** connecting to the anterior branch of fibular artery (52%) (Fig. 1) **Vascular density – 4.5%**
	**Type IB** – lateral anterior malleolar artery present and supplies anterior aspect of syndesmosis **directly** (16%) (Figs 2 and 3) **Vascular density – 5.8%**
	**Type IC** – Type IA or IB with coexisting anatomical variation of blood supply to posterior aspect of tibiofibular syndesmosis (4%) (Fig. 4) **Vascular density – 4.2%**
**Type II** (**28%**) Anterior aspect of tibiofibular syndesmosis supplied exclusively by anterior branch of fibular artery, with lack of lateral anterior malleolar artery	**Type IIA** – lack of lateral anterior malleolar artery but presence of anterior tibial artery (24%) (Fig. 5) **Vascular density – 3.8%**
	**Type IIB** – lack of anterior tibial and lateral malleolar artery (4%) (Fig. 6) **Vascular density – 3.5%**
	**Type IIC** – Type IIA or IIB with coexisting anatomical variation of blood supply to posterior aspect of tibiofibular syndesmosis (not obtained in our study – 0%) **Vascular density – N/A**

According to our observation, the most common type (I) was described in 36 cases (72%). Its characteristic feature was complete blood supply of anterior aspect of the syndesmosis by lateral anterior malleolar artery from anterior tibial artery and by anterior branch of fibular artery. Type I was divided into three subtypes: IA when lateral anterior malleolar artery was present and supplied anterior aspect of syndesmosis indirectly connecting to the anterior branch of fibular artery. It occurred in 26 cases (52%) (Fig. 1). In type IB – lateral anterior malleolar artery was present and supplied anterior aspect of syndesmosis directly. It occurred in 8 cases (16%) (Figs 2 and 3). Type IC which was defined as Type IA or IB with coexisting anatomical variation of blood supply to posterior aspect of tibiofibular syndesmosis was the rarest type and occurred in only 2 cases (4%) (Fig. 4).

Type II was found in 14 cases (28%). In this type, anterior aspect of tibiofibular syndesmosis was supplied exclusively by anterior branch of fibular artery, with lack of lateral anterior malleolar artery. Similarly to type I, type II was divided into three subtypes. In type IIA which was represented by 12 cases (12%), there was lack of lateral anterior malleolar artery but anterior tibial artery was present (Fig. 5). In type IIB found in 2 cases (4%), lack of anterior tibial and lateral malleolar artery was discovered (Fig. 6). We included type IIC in the classification however it was not obtained in our study (0%). It reflects the possible combination of type IIA or IIB with coexisting anatomical variation of blood supply to posterior aspect of tibiofibular syndesmosis.

According to our analysis, mean vascular density of tibiofibular syndesmosis was relatively low (4.4%) and closely correlated with the specific type of its blood supply according to the classification system introduced in this study. The percentage of combined lumen of the blood vessel was 4.5% in type IA, 5.8% in type IB, 4.2% in type IC, 3.8% in type IIA and 3.5% in type IIB. The highest density was observed in type IB and the lowest in type IIB (5.8% versus 3.5%, respectively).

## Discussion

Tibiofibular syndesmosis in majority of cases is supplied by three branches of two main arteries of the leg. Anterior aspect of tibiofibular syndesmosis is supplied by lateral anterior malleolar artery from anterior tibial artery together with anterior branch of fibular artery, whereas posterior aspect by posterior branch of fibular artery. In the following study this complex vascular anatomy was organized into clinically useful classification system along with examination of vascular density of this area. Awareness of various types of tibiofibular syndesmosis arterial blood supply is crucial for orthopedic surgeons who operate in the ankle region and radiology specialists for the anatomic evaluation of this commonly injured structure. Approximately 5% to 10% of all ankle sprains and 23% of ankle fractures involve trauma to the tibiofibular syndesmosis^12–19^. Despite the amount of syndesmotic injuries, there is a lack of presentations in literature of all possible variations of its fairly complex vascular anatomy. Attinger *et al*. in his study of utilization of angiosomes of the foot in order to salvage the limb presented, that anterior tibial and fibular arteries are connected by anterior branch of fibular artery and lateral anterior malleolar artery^20^. According to authors of this study, disruption of that connection can put the lateral ankle soft tissue at risk. In our study this kind of connection was observed in 56% of cases (in types IA and IC)^18^. McKeon *et al*. study seems to be the most complete description^5^. In this analysis anatomical variations of tibiofibular syndesmosis blood supply were divided into three main types. In type I (63% of cases) – anterior branch of fibular artery was the main source of blood supply, with occasional anastomoses with anterior tibial artery. This type corresponds to our type IA (52% of cases enrolled in our study). In the second pattern (21%) the fibular artery gave rise of multiple branches to the anterior ligaments of syndesmotic area and was supplemented by branches of a lesser caliber arising from the anterior tibial artery which supplied syndesmosis directly^5^. In the third pattern (16%) the anterior tibial artery supplied branches of a larger caliber than that of the branches from the perforating branch of the fibular artery^5^. Second and third pattern of McKeon *et al*. study are similar to our type IB (16% of cases)^5^. In the classification system presented in our study we did not take into consideration the caliber of the vessels, only the total density of vessels calculated as the percentage of combined blood vessel lumen in the syndesmosis. Observations presented in McKeon’s study are complement with our findings that blood supply to the posterior aspect of syndesmosis is rather constant. However due to the limited number of specimens, cases with predominantly posterior blood supply, as the consequence of the lack of branches supplying anterior aspect of the syndesmosis, were omitted in McKeon’s study. This aspect is described in our study and completes the description proposed by McKeon *et al*.^5^.

According to McKeon *et al*. the vascular supply to the posterior syndesmotic area originated completely from the fibular artery in 63% of cases^5^. In 37% of cases the posterior tibial artery also provided small branches to supply the posterior syndesmosis. According to our study blood supply to posterior aspect of tibiofibular syndesmosis was rather constant and was supplied by posterior branch of fibular artery in 96% of cases (types: IA, IB, IIA, IIB) and in 4% of cases by branch of posterior tibial artery (type IC). McKeon *et al*.^5^ reported that there were no specimens in which due to the lack of anterior tibial artery, the posterior tibial artery contribution was the dominant supply to the posterior syndesmotic region. According to our study branch from posterior tibial artery was dominant in 4% of cases (type IC). Our classification system which is complement with McKeon *et al*.^5^ corresponds well with syndesmotic vascular density (Table 1). Considering the complexity of tibiofibular syndesmosis blood supply, it was found that mean vascular density of this structure is surprisingly low (4.4%) and correlates with the specific anatomical variant. According to our classification, it was observed that with lack of anterior malleolar artery (type IIA and IIB) density of blood vessels was decreased, and in case of complete vasculature of the syndesmosis, density was the highest (Table 1).

The limited blood supply to a number of anatomic structures has been proposed and investigated as a factor of negative influence on healing and important to consider at the time of preoperative planning^13–17^. Diminished vascular density of tibiofibular syndesmosis may elongate healing time after injuries to the ankle, however publications describing vascular density of this region, especially in clinical context have not been found. Borrelli and Lashgari described the vascular supply to the lateral hindfoot to offer an anatomic explanation for high rate of wound complications of the corner of the flap with a lateral extensile incision for open reduction and internal fixation of calcaneal fractures^14^. Fractures in watershed areas of bone necessitate increased stabilization time and are more prone to surgical intervention in order for healing to occur. The fifth metatarsal bone fracture has been an ongoing area of study because of the known tendency for nonunion that is thought to stem from disruption of or limitations to the blood supply in the location of the fracture^16,17^. Similarly, insufficient vascular supply to an area of ligamentous injury, specifically the tibiofibular syndesmosis, may lead to delayed healing and increased rates of complications^5^.

According to the literature, during ankle fractures there is often a concomitant injury to the tibiofibular syndesmosis, which usually requires anatomical reduction and orthopedic stabilization for optimal functional recovery. Dingemans *et al*. reported that in approximately 20% of patients with ankle fractures, there is a concomitant injury to the tibiofibular syndesmosis which requires stabilization, usually with one or more syndesmotic screws^21^. Although screw fixation remains the most commonly used method of syndesmosis stabilization, still the method of fixation, number of screws, screw size, placement, number of cortices and finally screw removal are all controversial and currently under ongoing discussion among orthopedic surgeons^12,19,21,22^. Despite the amount of research devoted, many unanswered questions remain. Awareness of the degree of vascularization of tibiofibular syndesmosis may have an effect on whether to lengthen or shorten the duration of the syndesmotic screw stabilization. Moreover, according to the literature postoperative, routine hardware removal after screw fixation is being brought increasingly into question^19,21^. According to current knowledge this approach is not supported by evidence and may not be necessary. Increase in morbidity and substantial economic burden for healthcare system along with significant amount of time involved in this elective procedure do not justify this approach^19^. It is worth to consider that another surgical manipulation in the lateral ankle region can put syndesmosis blood supply at more risk which inevitably can result in poor functional recovery.

Knowledge of anatomical variations in tibiofibular syndesmosis vasculature may help surgeons to modify their surgical technique to avoid further deterioration of blood supply of this area with already low density of blood vessels. Awareness of low blood supply of tibiofibular syndesmosis may influence on treatment approach by elongating recommended time of syndesmotic screw stabilization or even retaining the hardware in order to prevent healing complications and avoid increase in morbidity.

Limitation of the study included the low number of enrolled specimens. It is clear that more dissections are needed to be done to describe all types of arterial variations in order to complete proposed classification system. Another limitation of the following study was utilization of only simple method of histological staining (hematoxylin and eosin) in order to obtain general estimation of vascular density. Although this approach was enough for general orientation in density of blood vessels in examined area, further analysis of syndesmotic composition is needed. As the next step authors would like to examine histologic composition of tibiofibular syndesmosis utilizing specific tissue staining for better visual differentiation in order to increase the accuracy of estimations and provide detailed structure of tibiofibular syndesmosis.

A clear understanding of arterial blood supply of the tibiofibular syndesmosis is essential for proper identification and avoidance of accidental injury of the arteries during procedures in the ankle region. Although fairly complex vasculature, the overall density of blood vessels in tibiofibular syndesmosis is low and is one of the factors which make surgical treatment of this area highly challenging.

## Material and Methods

The material for the following study was obtained from routine cadaveric dissections in the Anatomy Department and Forensic Medicine Department, Jagiellonian University Medical College, Krakow, Poland. We dissected 50 human ankles of both sexes in the age from 35 to 76, observing different types of tibiofibular syndesmosis arterial blood supply (Table 2). Immediately after amputation material was preserved by storage and injection of large vessels with formaldehyde solution for further dissection and analysis of vasculature. Preservation solution was prepared based on 8% formaldehyde diluted with distilled water. To limit the potentially negative effect of the chemicals on the integrity of small vessels, the dissection was completed and all histology specimens were obtained within the first week after amputation. Proper selection and approach to the preservation method eliminated significant impact on the results of the study. Three male and one female ankle were excluded from examination due to evident and visible macroscopic atherosclerotic changes, as an important factor which might have an influence on inconsistent results in macroscopic description of examined vessels and corresponding changes in microcirculation.

**Table 2:** Characteristics of studied lateral ankle specimens.

		Number	Percentage
Sex	Female	31	62%
	Male	19	38%

Access for proper visualization of the arterial blood supply of the tibiofibular syndesmosis was obtained by remove of the fibula. For this purpose a vertical incision on the posterior aspect of the leg was done, from the region of popliteal fossa to the plantar tendon. The incision was made along the axis of the bone, a finger breadth posterior to the fibula and was similar to the incision made in order to harvest fibular flap for reconstruction purposes. The skin, the muscles of posterior and lateral groups of the leg and a distal part of fibula were removed. The small vessels supplying lateral malleolar region were shown and described. Images of dissected specimens presented in this study, were recorded with digital camera (Canon PowerShot G9, 12MP) and then analyzed with Photoshop software (Adobe-Photoshop CS2, version 9.0.2.).

**Figure 7:**
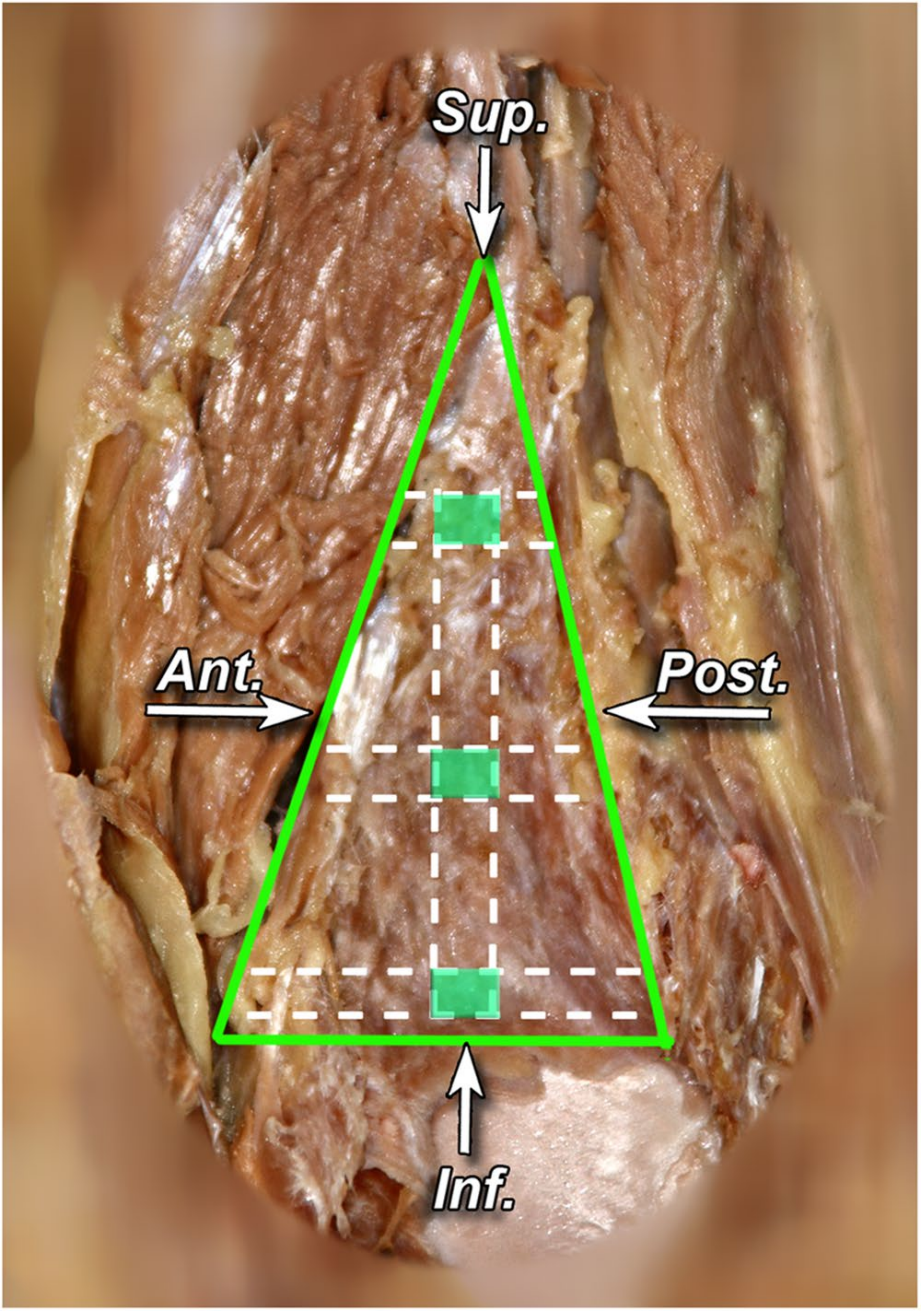
Sagittal section of tibiofibular syndesmosis presenting locations of cut out of small pieces of syndesmotic area – marked in red (left lower limb, preparation preserved in formaldehyde).

**Figure 8:**
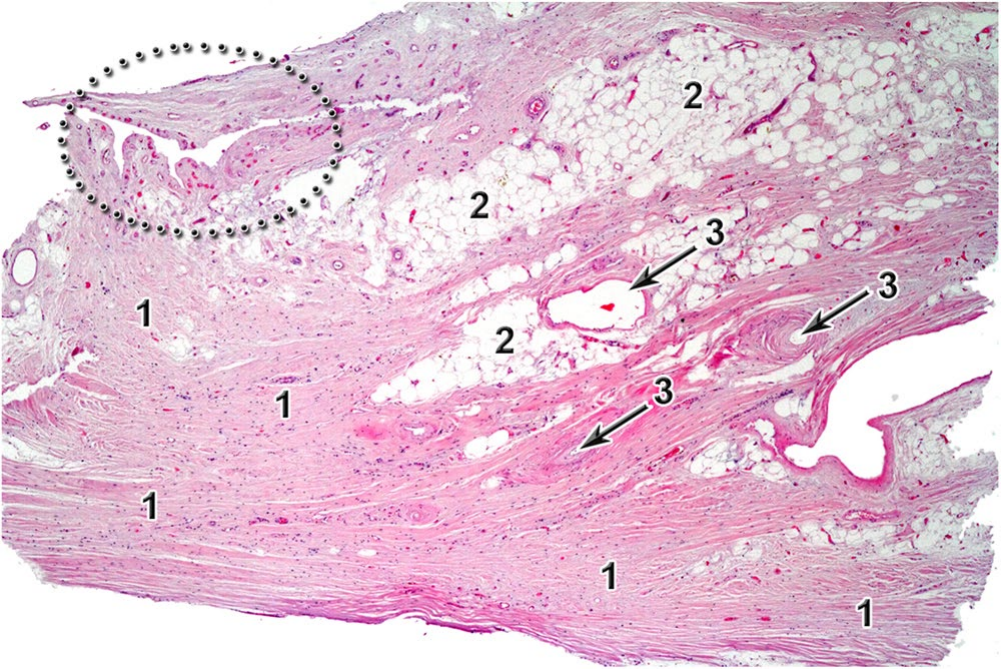
Histology image of tibiofibular syndesmosis, hematoxylin and eosine staining, 40x magnitude. 1 – fibrous tissue, 2 – adipose tissue, 3 – blood vessel lumen. In dark dotted area red dots represents small blood vessels.

In order to examine the vascular density of tibiofibular syndesmosis material was evaluated under microscope in the Department of Pathology, Jagiellonian University Medical College, Krakow, Poland. Three sections were taken from upper, middle and lower part of tibiofibular syndesmosis (Fig. 7). Tissue samples were sectioned parallel to their long axis – so the blood vessels lumen would be visualized perpendicular. The samples were dehydrated in ethanol, fixed in paraffin, sectioned to obtain 4 μm thick tissues slices, and stained with hematoxylin and eosine. The slides were assessed and photographed using a 5–40 magnification (Olympus BX43). The obtained images were analyzed using ImageJ software (Fig. 8). The percentage of fibrous tissue, adipose tissue, and the blood vessel combined lumen area to the whole area of the sample was analyzed per field of view. This percentage of combined vessel lumen to other tissues in syndesmosis per field of view represents estimation of its vascular density. The obtained values were averaged across the samples, stratifying for age.

Regarding the experiments involving human participants (including the use of tissue samples) we have obtained informed consent for study participation and approval from the Jagiellonian University Medical College Bioethics Committee (registry no. KBET/167/B/2009) for routine cadaveric dissections in the Anatomy Department and Forensic Medicine Department, Jagiellonian University Medical College, Krakow, Poland. This study adhered to the Declaration of Helsinki and its later amendments. Due to the limited number of enrolled specimens statistical analysis was withdrawn.
